# Chemical Composition and Structural Traits of Leaf Biomass in Selected Asparagaceae Species

**DOI:** 10.3390/plants15030468

**Published:** 2026-02-02

**Authors:** Nadia Villada-Lozada, Agustina Rosa Andrés-Hernández, Agustín Maceda

**Affiliations:** Plant Anatomy Laboratory, Biology Faculty, Benemérita Universidad Autónoma de Puebla, Blvd. Valsequillo y Av. San Claudio, Edificio 112-A, Ciudad Universitaria, Col. Jardines de San Manuel, Puebla, Puebla 72570, Mexico; nadia.villada@alumno.buap.mx

**Keywords:** Asparagaceae, chemical composition, lignocellulosic components, FTIR spectroscopy, anatomical traits, biomass utilization

## Abstract

This study presents an integrated chemical and anatomical characterization of leaves from seven Asparagaceae species (*Agave convallis* Trel., *A. salmiana* Otto ex Salm.-Dyck, *A. striata* Zucc., *Dasylirion acrotrichum* Zucc., *Nolina excelsa* García-Mend. & E. Solano, *Yucca filifera* Chabaud, and *Y. periculosa* Baker). Leaf biomass was subjected to successive Soxhlet extractions to quantify extractives, followed by isolation of lignocellulosic fractions. Lignin and cellulose were analyzed by Fourier-transform infrared (FTIR) spectroscopy to determine the syringyl/guaiacyl (S/G) ratio and total crystallinity index. Leaf anatomy was examined using fluorescence microscopy. Total extractives ranged from 13.4 to 24.0%, with *A. salmiana* and *D. acrotrichum* showing the highest values. Lignin content varied markedly among genera, reaching up to 45.1% in *Yucca* species, whereas cellulose content ranged from 31.3 to 42.2%. Crystalline cellulose accounted for 42.1–56.9% of total cellulose, with the highest crystallinity observed in *A. convallis*. FTIR analysis revealed a predominance of guaiacyl-type lignin in all species except *Y. periculosa* (S/G = 1.2). Multivariate analyses discriminated between genera primarily based on lignin, hemicellulose, and cellulose contents. These findings highlight genus-level differences in leaf lignocellulosic composition and support the potential use of Asparagaceae leaves as feedstocks for bioenergy and biomaterial applications.

## 1. Introduction

The family Asparagaceae comprises more than 2900 species with a wide global distribution [1]. In Mexico, its representatives are mainly found in arid and semi-arid regions, where they have evolved a range of adaptations to water stress. These include leaf and stem succulence, thick cuticles that reduce desiccation, and abundant parenchyma specialized in water storage [2,3]. Such anatomical traits have been documented in economically important species such as *Agave tequilana* F.A.C.Weber and *A. fourcroydes* Lem., which also possess specialized cells for water and mucilage storage [4]. Genera exhibiting these adaptations and notable for their high levels of endemism in Mexico include *Agave*, *Dasylirion*, *Nolina*, and *Yucca* [3]. Ecologically, these species contribute to soil conservation, provide resources for pollinators, and offer shelter for diverse animal species [5].

In addition to their ecological relevance, Asparagaceae species are of considerable economic importance. *Agave* species have been widely exploited for fiber production, forage, and the manufacture of fermented and distilled beverages such as tequila and mezcal [6]. Some *Dasylirion* species are used for sotol production, whereas *Yucca* and *Nolina* species have traditionally been employed in folk medicine and construction materials [7].

The fibrous nature of agave leaves [8,9] enables their use in material production, while structural components such as lignin and cellulose are key resources for the development of biofuels and lignocellulosic biomaterials, including paper [10]. Lignin, together with cellulose, is a major constituent of the secondary cell wall and plays essential roles in mechanical support, water transport, and defense against biotic and abiotic stresses [11]. From an evolutionary and functional perspective, lignin composition varies among plant groups, with guaiacyl- and syringyl-type monomers predominating. Syringyl-rich lignin, characterized by a higher proportion of β-O-4 linkages, is generally more susceptible to chemical degradation [12].

Crystalline cellulose is the principal structural polysaccharide of the plant cell wall, and its abundance and organization are closely linked to tissue rigidity and conductive efficiency. Cellulose occurs in both crystalline and amorphous forms (Marinho, 2025), with its main accumulation in primary cell walls, while the relative proportions of cellulose and lignin vary during secondary wall development [13].

Most studies on Asparagaceae have focused on economically important *Agave* species, such as *A. angustifolia* Haw., *A. americana* L., *A. salmiana* Otto ex Salm-Dyck, and *A. tequilana* F.A.C.Weber, addressing leaf and fiber extractives, cellulose structure, and syringyl/guaiacyl (S/G) ratios [8,14,15,16,17,18]. In contrast, fewer studies have examined other genera, such as *Yucca*, *Nolina*, and *Dasylirion*, particularly regarding leaf lignocellulosic composition and crystalline structure [19,20]. Although some analyses have addressed stem structure across several genera [3,21], stem utilization results in plant death, making leaves a more sustainable resource.

Moreover, most Asparagaceae species exhibit crassulacean acid metabolism (CAM) and pronounced morphoanatomical adaptations to drought [22,23]. Consequently, their leaves represent a promising source of second-generation biofuels and lignocellulosic materials that do not compete with staple food crops [18,24,25].

Given that most studies within Asparagaceae have focused on economically important cultivated species, while wild or less-utilized taxa remain poorly characterized, the present study aimed to chemically and anatomically characterize the leaves of seven Asparagaceae species from Mexico to identify their structural traits and potential uses. We hypothesized that leaves of Asparagaceae species exhibit genus-specific differences in extractives and lignocellulosic composition that are closely associated with leaf anatomy and adaptations to water stress, with a predominance of crystalline cellulose and guaiacyl-type lignin conferring structural support and functional resilience under arid and semi-arid conditions.

## 2. Results

### 2.1. Extractives and Lignocellulosic Composition

The chemical composition of leaves differed markedly among the seven Asparagaceae species (Table 1). For ethanol:toluene extractives, the lowest percentages were observed in both *Yucca* species, whereas *Dasylirion acrotrichum* showed the highest proportion of non-polar extractives. For ethanol extractives, *Agave salmiana* exhibited the highest value while the remaining species ranged from 0.92 to 2.62%.

Regarding hot-water extractives, *Nolina excelsa* García-Mend. & E. Solano showed the lowest percentage, whereas *Yucca filifera* Chabaud exhibited the highest value. Total extractives ranged from 13.37 to 24% with *Agave salmiana* and *Dasylirion acrotrichum* Zucc. showing the highest values, whereas *Nolina excelsa* presented the lowest extractives content. This variability indicates substantial interspecific differences in non-structural compounds associated with secondary metabolism. Differences in extractives content may also influence biomass processing, as non-structural compounds can affect cell wall accessibility and downstream chemical analyses. Therefore, variation in extractives contributes not only to interspecific chemical diversity but also to the functional classification of leaf biomass among the studied species.

Lignin content showed pronounced genus-level patterns. *Yucca* species exhibited the highest lignin proportions, reaching up to 42.79% in *Yucca periculosa* Baker, whereas *Agave* species displayed comparatively lower lignin values (28.57–32.12%). Hemicellulose content varied within a narrower range (23.40–36.32%), but contributed to differentiation between genera, particularly between *Agave* and *Yucca*. Cellulose content ranged from 31.35 –42.15%, with *Agave convallis* Trel. and *A. striata* Zucc. presenting the highest values, whereas *A. striata* (31.56%) and *Y. filifera* (31.35%) showed the lowest values (Table 1).

### 2.2. Crystalline Cellulose Index

**Figure 1:**
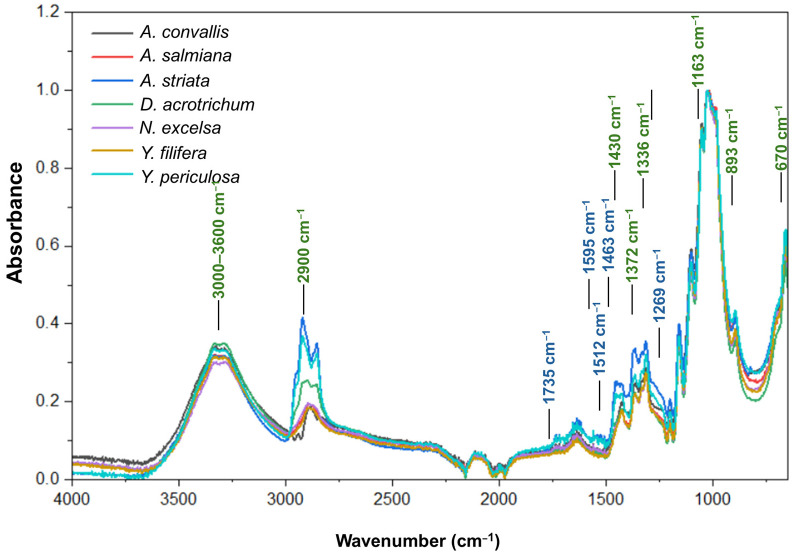
FTIR spectra of purified cellulose obtained from leaves of the studied Asparagaceae species. The main absorption bands corresponding to cellulose functional groups are indicated, including O–H stretching (3000–3600 cm^−1^), C–H stretching (~2900 cm^−1^), CH_2_ symmetric bending (1430 cm^−1^), and C–O–C asymmetric stretching (1163 cm^−1^). Bands associated with hemicelluloses (1735 and 1269 cm^−1^) are absent, indicating effective cellulose purification. Spectra correspond to average measurements.

Based on FTIR spectra obtained from purified cellulose samples, the intensities of characteristic cellulose bands were clearly identified, together with the absence of absorption bands associated with xylans and hemicelluloses at 1735 cm^−1^ and 1269 cm^−1^. Minor traces of lignin were detected, as indicated by weak band intensities at 1595, 1512, and 1463 cm^−1^ (Figure 1).

The principal absorption bands of the cellulose were detected in the FTIR spectra and assigned as follows: O–H stretching vibrations in the 3000–3600 cm^−1^ region, C–H stretching at ~2900 cm^−1^, CH_2_ symmetric bending at 1430 cm^−1^ (associated with both amorphous and crystalline cellulose), C–H and C–O bending vibrations at 1372 cm^−1^, C–O–H in-plane bending at 1336 cm^−1^ (amorphous cellulose), CH_2_ wagging vibrations at 1315 cm^−1^ (crystalline cellulose), C–O–C asymmetric stretching at 1163 cm^−1^, out-of-plane asymmetric stretching of the cellulose ring at 893 cm^−1^, and C–O–H out-of-plane stretching at 670 cm^−1^ (Figure 1).

Crystalline cellulose percentages were heterogeneous, even within the same genera (*Agave* and *Yucca*) (Table 2). Species with TCI values below 1 were those in which the presence of amorphous cellulose predominated in the cell walls, while above 1, there is a greater presence of crystalline cellulose in the cell walls of the leaves. *Agave convallis* showed the highest crystalline cellulose content, followed by *Yucca filifera* and *A. salmiana*. The lowest values were recorded for *Y. periculosa* (Table 2).

### 2.3. Lignin Characterization

**Figure 2:**
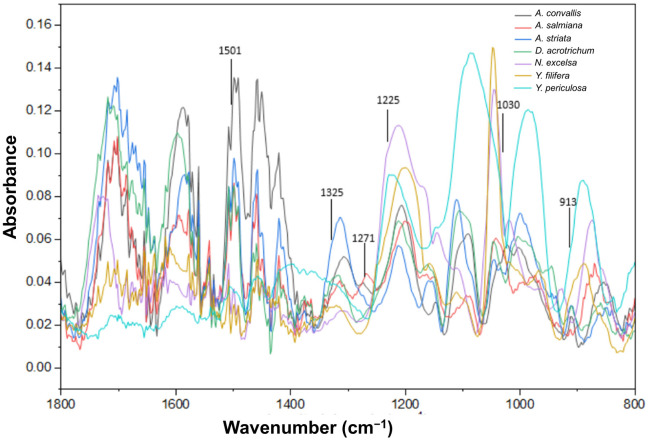
FTIR spectra of purified lignin isolated from leaves of the studied Asparagaceae species. Characteristic absorption bands associated with syringyl and guaiacyl lignin units are shown, including aromatic ring vibrations (~1501 cm^−1^), syringyl-associated bands (1325 cm^−1^), and guaiacyl-associated bands (1271 cm^−1^). Relative band intensities were used for the calculation of the syringyl/guaiacyl (S/G) ratio.

FTIR spectra of purified lignin samples showed the characteristic lignin absorption bands (Figure 2). The band at 913 cm^−1^ corresponded to =CH out-of-plane deformation in the aromatic rings of syringyl and guaiacyl monomers. The band at 1030 cm^−1^ was associated with C–H in-plane deformation of guaiacyl units and C–O deformation in primary alcohols, whereas the bands at 1271 and 1225 cm^−1^ were assigned to vibrations of guaiacyl and syringyl units, including symmetric C–O stretching. The band at 1325 cm^−1^ was attributed to ring breathing of syringyl units and C–O stretching, while the band at 1501 cm^−1^ corresponded to aromatic C=C ring vibrations of syringyl and guaiacyl monomers (Figure 2).

Band intensities at 1325 and 1271 cm^−1^ were used to calculate the syringyl/guaiacyl (S/G) ratio (Table 3). All species exhibited a predominance of guaiacyl units (50.7–54.7%), except for *Yucca periculosa*, which showed a higher syringyl contribution (54.6%), resulting in an S/G ratio of 1.2. The lowest S/G ratio was observed in *Agave convallis* (Table 3).

### 2.4. Principal Component Analysis

Based on the principal component analysis (PCA), the three PCs presented eigenvalues greater than 1 and were therefore relevant for explaining species separation. The variables with the highest positive or negative loadings in each PC were responsible for species differentiation. In PC1, lignin and hemicellulose content were the variables that most strongly influenced species separation (Table 4). In PC2, extractive-free lignocellulose and cellulose were the determining variables, whereas in PC3, ethanol:toluene and hot-water extractives were the main contributors (Table 4).

**Figure 3:**
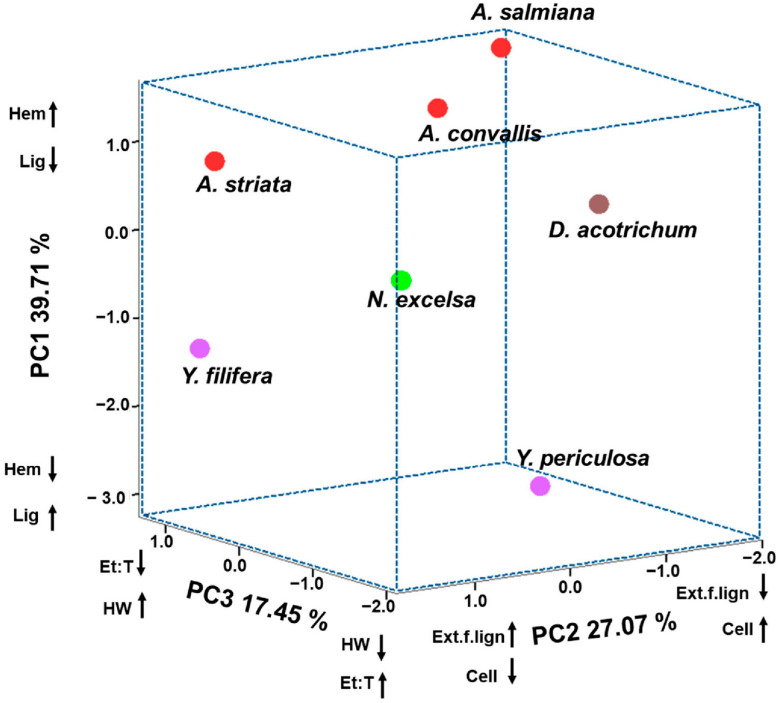
Three-dimensional PCA scatter plot based on the chemical composition and anatomical characteristics of the leaves of seven Asparagaceae species. Percentages indicate the proportion of variance explained by each principal component. Cell: cellulose. Et:T: Ethanol:toluene. Ext.f.lign: Extractives free lignocellulose. Hem: hemicellulose. Lig: lignin. HW: Hot water. Arrows indicate the direction of increasing (↑) or decreasing (↓) values of the variables associated with each principal component.

Using the three PCs and the variables driving species dispersion, a score plot was generated showing clear separation among the seven species (Figure 3). *Agave* species were located on the positive side of PC1, as they exhibited lower lignin content and higher hemicellulose percentages, whereas *Yucca* species showed higher lignin content in their leaves. *Nolina excelsa* and *D. acrotrichum* occupied intermediate positions, reflecting similar lignin and hemicellulose proportions.

Along PC2, variation in cellulose and extractive-free lignocellulose content was observed within both *Agave* and *Yucca*. *Yucca periculosa*, *D. acrotrichum*, *A. convallis*, and *A. salmiana* exhibited higher cellulose content and lower extractive-free lignocellulose, whereas *Y. filifera* and *A. striata* showed lower cellulose content and higher extractive-free lignocellulose values (Figure 3; Table 4).

In PC3, species with lower ethanol:toluene extractives and higher hot-water extractives included the three *Agave* species and both *Yucca* species. In contrast, *N. excelsa* and *D. acrotrichum* exhibited higher ethanol:toluene extractives and lower hot-water extractives. Overall, the multivariate analysis confirmed that lignin proportion, cellulose crystallinity, and extractives content were the main variables explaining species and genus discrimination, integrating chemical and structural features of leaf biomass.

### 2.5. Anatomical Distribution of Lignocellulose

**Figure 4:**
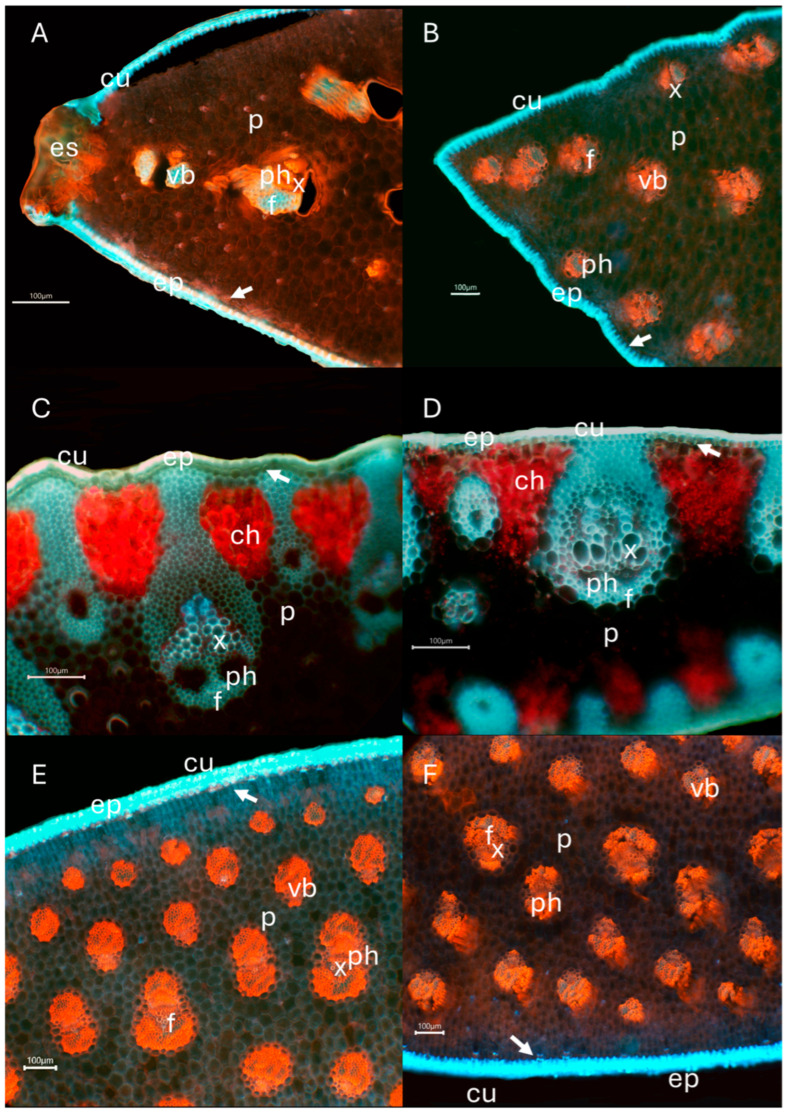
Fluorescence microscopy images of transverse leaf sections from representative Asparagaceae species showing the spatial distribution of lignified and non-lignified tissues. (**A**) *Agave convallis*; (**B**) *Agave striata*; (**C**) *Dasylirion acrotrichum*; (**D**) *Nolina excelsa*; (**E**) *Yucca filifera*; (**F**) *Yucca periculosa*. Panels (**A**,**B**,**E**,**F**) correspond to sections stained with Congo red and acridine orange, whereas panels (**C,D**) show unstained sections observed under natural autofluorescence. Fluorescence signals were recorded using three excitation/emission filter sets: DAPI (excitation 365 nm, emission 445/50 nm), FITC (excitation 470/40 nm, emission 525/50 nm), and TRITC (excitation 546/12 nm, emission 575–640 nm). Different staining and imaging conditions were intentionally applied to facilitate anatomical observation of tissue organization and the distribution of lignified tissues, rather than for direct quantitative comparison among samples. White arrows indicate the hypodermis. Abbreviations: ch, chlorenchyma; cu, cuticle; ep, epidermis; es, sclereids; f, fibers; ph, phloem; p, parenchyma; x, xylem; vb, vascular bundle. Scale bars = 100 µm.

In all species, the main leaf characteristic was the presence of abundant non-lignified parenchyma, except in *Dasylirion acrotrichum* and *Nolina excelsa*, which showed a higher proportion of fibers and lignified parenchyma. In *Agave* and *Yucca* species, vascular bundles were isolated and separated by parenchyma, whereas in *D. acrotrichum* and *N. excelsa*, vascular bundles were connected to a lignified hypodermis (Figure 4).

All species exhibited a thick cuticle on the outer epidermal wall. In *Agave*, the hypodermis was not lignified, whereas in the remaining species, lignin accumulation was evident. Lignin emitted green to bluish fluorescence in all species, particularly in vascular bundles, while cellulose emitted red fluorescence. Crystalline cellulose showed higher fluorescence intensity than cellulose with a predominantly amorphous structure (Figure 4).

The chemical patterns described above were consistent with anatomical observations. Species with higher lignin content, particularly *Yucca* spp., showed a greater proportion of lignified tissues and more heavily lignified vascular bundles. In contrast, *Agave* species, characterized by higher cellulose content and crystallinity, exhibited abundant non-lignified parenchyma and isolated vascular bundles embedded in succulent tissue. The observed differences reflect variation in chemical composition and structural traits among species.

Vascular bundles of *Agave* and *Yucca* exhibited greater cellulose accumulation than those of *D. acrotrichum* and *N. excelsa*, in which lignin accumulation predominated. Xylem vessels showed lignified secondary walls, whereas phloem tissues exhibited cellulose-rich walls with red fluorescence in samples stained with Congo red. In *D. acrotrichum* and *N. excelsa*, which were examined by autofluorescence, no cellulose fluorescence was observed, as cellulose does not autofluoresce.

Chlorenchyma was evident in *D. acrotrichum* and *N. excelsa*, allowing for the visualization of chloroplasts surrounding vascular bundles, whereas no chloroplasts were observed in the central leaf tissue.

## 3. Discussion

Leaves of the genera *Agave*, *Dasylirion*, *Nolina*, and *Yucca* showed differences in the percentages of extractive compounds and in each of the lignocellulosic components. Differences were also observed in the crystalline or amorphous structure of cellulose and in the proportion of syringyl and guaiacyl units that compose lignin. These chemical differences were consistent with the leaf anatomy observed across all species.

### 3.1. Extractives

All analyzed variables of extractives showed significant differences among species. Asparagaceae species were heterogeneous in the accumulation of extractives across solvents of different polarity. The first extraction, corresponding to ethanol:toluene, yielded the highest values compared to the other two extractive types. This is because non-polar solvents extract a wide variety of lipids, oils, waxes, paraffins, organic acids, resins, and sulphones [26,27,28].

Extractives obtained with 96% ethanol showed the lowest percentages overall, ranging from 0.92 to 4.62%. This extraction primarily recovers polar compounds, most of which correspond to gums, tannins, salts, carbohydrates, terpenoids, and phenolic compounds [28,29]. Hot-water extractives were higher than those obtained with 96% ethanol, with values ranging from 2.46 to 8.21%. The compounds solubilized in this extraction are fully polar and include minerals, salts, gums, tannins, and pigments [28,30].

Overall extractive percentages were higher than those reported for *Agave* species. For ethanol:toluene extractives, values of 2.9–3.7% have been reported for *Agave tequilana* fibers and bagasse [9,31,32], 1.5% for fibers of *A. angustifolia* Adrian Hardy Haworth [8], and 4% for leaves of *A. lechuguilla* Torr. [33]. These differences are likely since the previously reported species were cultivated under specific conditions, whereas the species analyzed here are practically wild. Environmental conditions strongly influence the accumulation of these metabolites [34].

For 96% ethanol extractives, reported values include 1.3% for leaves of *A. americana* [14] and 5% for leaves of *A. lechuguilla* [33], which are similar to the values obtained in this study. Regarding hot-water extractives at 90 °C, values of 6% have been reported for leaves of *A. americana* L. [14], 4.4% for fibers of *A. angustifolia* [8], and 3.7–10.6% for bagasse and fibers of *A. tequilana* Weber [9,31,32]. For leaves of *A. lechuguilla*, values of 4% have been reported [33]. These percentages were heterogeneous among species, with some values lower and others like those reported here.

Total extractives reported for *A. angustifolia* fibers range from 2.5 to 5.3% [8,35], for stems of *A. lechuguilla* from 25.7 to 45.3% [36,37], and for fibers of *Yucca gloriosa* 1.1% [19]. These highly variable percentages are likely related to environmental conditions, the plant structure analyzed (fibers, bagasse, or leaves), and species-specific traits. For species of the genera *Dasylirion*, *Nolina*, and *Yucca*, limited information is available, mainly for stems, as reported by Maceda et al. [3], who found total extractive contents similar to those reported here, ranging from 11% (*Nolina excelsa*) to 24.1% (*Yucca gigantea*). Therefore, future studies should focus on the chemical characterization of extractable compounds from these species, as compounds from *Agave* species have shown antimicrobial activity [38] and potential applications in the food and pharmaceutical industries [39,40].

Extractives content represents an additional dimension influencing the functional classification of Asparagaceae leaf biomass. Although not structurally integrated into the cell wall, extractives can affect processing efficiency, chemical accessibility, and the recovery of value-added compounds [40]. In the present study, species with higher extractives levels, particularly *Agave salmiana* and *Dasylirion acrotrichum*, contributed to multivariate separation and may offer opportunities for co-product valorization alongside lignocellulosic applications. Conversely, species with lower extractives content, such as *Nolina excelsa*, may favor more homogeneous lignocellulosic processing. Thus, extractives complement lignin and cellulose traits in defining integrated biomass profiles rather than representing isolated chemical features.

### 3.2. Lignocellulose and Principal Components

Table S1 (Supplementary Material) summarizes lignocellulosic percentages reported for other Asparagaceae species. In general, cellulose content varies widely among genera, with values ranging from 79.8% (*A. lechuguilla*) to 24.7% (*A. tequilana*) (Table S1). *Agave striata*, *D. acrotrichum*, *N. excelsa*, and *Yucca* spp. showed values between 31.56 and 35.06%, like those reported for *A. tequilana*, *A. americana*, *A. salmiana*, and *A. lechuguilla* [14,41,42]. In contrast, *A. convallis* and *A. salmiana* (41.09 and 42.15%, respectively) showed values comparable to *A. sisalana* (43%) [43], *A. tequilana* (42%) [44,45], and *A. lechuguilla* and *A. salmiana* (44.5–45%) [14,41,42] (Table S1).

For other Asparagaceae species, cellulose percentages in leaves were generally lower than those reported in most studies, likely due to differences in purification methods [46] and because extractive-free lignocellulose was analyzed here, whereas previous studies focused on fibers and bagasse [47]. Nevertheless, the values obtained indicate that leaves could be used as a cellulose source for the paper industry [48].

Lignin percentages reported in Table S1 range from 2.86% in *A. lechuguilla*, *A. salmiana*, and *A. tequilana* [42] to 25.3% in *A. tequilana* [32], values that are comparable to or lower than those reported here. The higher lignin content observed in the leaves analyzed in this study may be related to the fact that these species are wild and grow under water-stress conditions, whereas most species listed in Table S1 are cultivated [49,50]. Elevated lignin content also highlights the potential of these leaves as carbon reservoirs and as biomass for energy production through combustion [51].

Hemicellulose content also varies among and within species [52], with reported values ranging from 3 to 34.08% (Table S1). In this study, values ranged from 23.4 to 36.32%. High hemicellulose content can be exploited to produce biofuels and bioproducts [53].

From a lignocellulosic perspective, the observed variation in cellulose, hemicellulose, and lignin content defines distinct functional biomass profiles among the studied species. Higher cellulose proportions, as observed in *Agave* species, are commonly associated with increased structural polysaccharide availability and suitability for fiber-based or cellulose-derived applications [10]. In contrast, species characterized by higher lignin content, particularly *Yucca* spp., exhibit chemical features linked to greater structural rigidity and biomass recalcitrance, traits often considered advantageous for lignin-oriented or bioenergy-related uses [12]. These differences highlight complementary lignocellulosic strategies rather than hierarchical suitability, indicating that Asparagaceae leaves may serve as non-food biomass resources tailored to different utilization pathways depending on their chemical composition [7].

Principal component analysis (PCA) showed that genera clustered according to similarities in extractive and lignocellulosic content. However, the variables showing the greatest variation within genera were extractive-free lignocellulose, cellulose, ethanol:toluene extractives, and hot-water extractives. As reported for other succulent species such as Cactaceae [54] (Maceda et al., 2018), lignin, hemicellulose, and cellulose contents were the main drivers of intergeneric separation, whereas extractives explained intrageneric variation. Differences in lignocellulose and extractive accumulation are mainly related to plant adaptations to different environments, while succulence and secondary metabolite accumulation are associated with chemical defense mechanisms [55].

The multivariate separation observed in the PCA is not only taxonomic but also functional, as it reflects distinct lignocellulosic biomass profiles with potential application relevance. Species grouped along PC2, characterized by higher cellulose content and crystallinity, particularly *Agave* spp., display features desirable for fiber-based materials and cellulose-derived products, where ordered cellulose structure is a key requirement.

In contrast, species separated along PC1, mainly driven by higher lignin content, such as *Yucca* spp., exhibit chemical traits commonly associated with bioenergy applications and lignin-rich materials. The intermediate positioning of *Dasylirion acrotrichum* and *Nolina excelsa* suggests a combination of chemical composition and structural traits that may be suitable for multipurpose biomass utilization.

Therefore, PCA serves as an effective integrative tool to classify Asparagaceae species according to their functional lignocellulosic profiles, providing a framework for selecting non-food plant resources based on targeted biotechnological applications rather than solely on taxonomic criteria.

### 3.3. Crystalline Cellulose and S/G Lignin

The crystallinity index of Asparagaceae leaves ranged from 0.73 to 1.32. Four species showed crystalline cellulose contents above 50%, whereas three species ranged from 42.1 to 46.9%, indicating heterogeneity in crystalline cellulose accumulation. These values are comparable to those reported for leaves of *A. americana* (45–55%) [14], fibers of *A. salmiana* (50.07%) [15], fibers of *Yucca* spp. (55–56%) [56], and *Furcraea foetida* (52.6%) [57]. Higher cellulose contents (60.5–76%) have been reported mainly for fibers and bagasse of Agave [32,47,58] and Yucca species [20,59].

Higher cellulose accumulation has also been reported for stems of several Asparagaceae species [3], with values similar to those obtained here. Species with higher crystalline cellulose content may be suitable to produce microfibrillated cellulose nanofibers used in medical devices [60,61], materials engineering, and paper recycling processes, where cellulose provides structural reinforcement [62].

Few studies have reported syringyl/guaiacyl (S/G) ratios for Asparagaceae. Maceda et al. [3] reported that, in stems of 10 species, eight exhibited S/G ratios above 1 and up to 3.9, indicating a predominance of syringyl units. This contrasts with the present study, where only *Y. periculosa* showed an S/G ratio of 1.2.

According to Li et al. [63], syringyl accumulation is associated mainly with fiber-rich tissues. However, although leaves contain fibers, guaiacyl units predominated, supporting findings by Maceda et al. [12] for Cactaceae and Asparagaceae, where syringyl units in succulent stems act as protection against pathogen attack [64]. In Asparagaceae, syringyl accumulation in stems may be related to their higher sugar content compared to leaves [65,66], providing not only structural support but also pathogen defense. In contrast, leaves show lower sugar content at the tip, higher content in the middle, and the highest content at the base, with fructans being the predominant sugars, which are more resistant to bacterial degradation [65]. Therefore, guaiacyl units likely contribute to leaf rigidity and mechanical support.

In addition to their structural significance, cellulose crystallinity and lignin monomer composition have important implications for biomass utilization. Higher cellulose crystallinity, as observed in certain *Agave* species, is commonly associated with increased mechanical stability and resistance to chemical disruption, traits relevant for fiber-based materials and structural applications [62]. Conversely, variation in the lignin syringyl/guaiacyl (S/G) ratio influences cell wall architecture and chemical reactivity, with higher guaiacyl content generally linked to more condensed lignin networks, as observed in most studied species, whereas increased syringyl contribution in *Yucca periculosa* suggests a less cross-linked lignin structure.

These differences in crystalline organization and lignin composition contribute to the functional differentiation revealed by multivariate analyses, reinforcing the value of integrating crystallinity and S/G ratio as descriptors of lignocellulosic biomass quality rather than purely compositional parameters.

### 3.4. Anatomical Distribution

Leaf anatomy was typical of the Asparagaceae family, characterized by vascular bundles arranged in patches, thick cuticles, lignified epidermis and hypodermis, and parenchyma specialized for water storage. These features have been reported for other *Agave* species [67,68] and for other genera within the family, such as *Dracaena* [69], *Manfreda* [70], and *Polianthes* [71].

The main anatomical differences among species were related to the proportion of non-lignified parenchyma, which was higher in *Agave* and *Yucca* than in *Nolina* and *Dasylirion*, where lignified parenchyma predominated. These differences were consistent with the cellulose and lignin percentages obtained for these genera.

Higher cellulose fluorescence intensity in vascular bundles reflects reinforcement of xylem walls and surrounding fibers, providing greater structural support to primary walls. In contrast, non-lignified parenchyma exhibited lower fluorescence, allowing for wall flexibility for water storage during rainy periods and dehydration during dry seasons without cell wall rupture. This trait has been reported for other succulent families [12] and represents a strategy to prevent collapse of conductive elements and parenchyma [72]. Crystalline cellulose also reinforces cell walls against enzymatic degradation by cellulases [73].

The general accumulation of guaiacyl units in leaves contributes to structural rigidity and prevents vascular bundle rupture. The presence of fiber patches surrounding xylem and phloem and adjacent to the epidermis enhances resistance to fracture and supports leaf weight. Due to the molecular structure of guaiacyl monomers, chemical degradation of fibers is challenging [74], requiring biological degradation processes [75] or their use in textile fiber development [10].

Beyond descriptive differences, the anatomical distribution of lignified and non-lignified tissues has direct implications for biomass accessibility and potential utilization. Species characterized by abundant non-lignified parenchyma and isolated vascular bundles, particularly *Agave* spp., may facilitate cellulose accessibility during mechanical or chemical processing, favoring fiber-based or cellulose-derived applications. In contrast, species with a higher proportion of lignified tissues and more compact vascular organization, such as *Yucca* spp., present anatomical features commonly associated with increased recalcitrance, which may be advantageous for lignin-rich materials or bioenergy-oriented uses. Thus, anatomical distribution complements chemical composition in defining functional lignocellulosic profiles among Asparagaceae species.

The combination of abundant non-lignified parenchyma, high extractive and lignocellulosic content, predominance of crystalline cellulose, and guaiacyl-type lignin indicates that leaves of the seven Asparagaceae species analyzed here have potential applications in biofuel production and biomaterials industries. Furthermore, as drought- and heat-tolerant species, they could be exploited in the development of new crops with diverse applications, while also improving our understanding of the physiological adaptations and cell wall composition of these organisms.

## 4. Materials and Methods

### 4.1. Plant Material

Leaves of seven—*Agave convallis* Trel., *A. salmiana* Otto ex. Salm-Dyck, *A. striata* Zucc., *Dasylirion acrotrichum* Zucc., *Nolina excelsa* García-Mend. & E.Solano, *Yucca filifera* Chabaud, and *Y. periculosa* Baker—were obtained from plants propagated vegetatively by stolons and donated by the Botanical Garden of the Universidad Nacional Autónoma de México (UNAM) (Table 5). The plants were originally collected from the Botanical Garden (see Table S2 for collection numbers), located at 19°18′44″ N, 99°11′46″ W, at 2320 m a.s.l. The area has a temperate climate with summer rainfall, a rainy season from June to October, a mean annual precipitation of 833 mm, and an average annual temperature of 15.6 °C.

Three mature individuals per species were analyzed. For each individual, leaves were collected from the middle portion of the stem to minimize age-related variability, avoiding both young central leaves and senescent basal leaves. Due to differences in leaf arrangement among species, this standardized positional criterion was applied consistently across all taxa. All collected leaves were visibly healthy and free of insect damage or mechanical injury. For each species, ten leaves were collected in triplicate. Two leaves were randomly selected, and the middle portion of each leaf was excised and fixed in formaldehyde–acetic acid–ethanol (FAA) solution for at least one week for anatomical analysis. The remaining leaves were used for chemical analyses, excluding the leaf tips, which contain a high lignin content.

For the chemical analysis, leaves were cut into 2 × 2 cm fragments and oven-dried at 70 °C for one week. Dried samples were ground using a Wiley mill (40–60 mesh; Thomas Scientific, Swedesboro, NJ, USA) to obtain a particle size of approximately 0.4 mm. All analyses were performed in triplicate following the TAPPI T−222 om−02 standard and based on methods described by Maceda et al. [12] and Reyes-Rivera and Terrazas [76].

From the ground material, 2 g were placed into pre-weighed filter paper thimbles and subjected to successive Soxhlet extractions for 6 h. The first extraction was performed using ethanol–benzene (1:2, *v*/*v*), followed by ethanol (96%). After each extraction, samples were dried at 60 °C for 24 h to constant weight. Subsequently, samples were boiled in distilled water at 90 °C for 1 h, dried at 80 °C for 24 h, and weighed.

Total extractives were calculated using the following equation:

*Total extractives* (%) = [(A + B + C/W_0_] × 100 (1)

where A is the mass loss after ethanol–benzene extraction, B is the mass loss after ethanol extraction, C is the mass loss after hot water extraction, and W_0_ is the initial dry weight of the sample. Extractive-free lignocellulose was calculated by subtracting total extractives from the initial dry weight.

### 4.2. Lignin Purification

Extractive-free lignocellulose was homogenized and finely ground using a mortar. For each species, 0.5 g of material were treated with 7.5 mL of 72% sulfuric acid at 2 °C and maintained under constant agitation at room temperature (18 °C) for 2 h. Subsequently, 350 mL of distilled water were added, and samples were boiled for 4 h at constant volume to hydrolyze holocellulose.

Samples were filtered through pre-weighed fine-pore Büchner filters and rinsed with 100 mL of distilled water. Filters containing lignin were oven-dried at 90 °C for 24 h to constant weight. Klason lignin content was calculated as:

*Klason lignin* (%) = (W_L_/W_W_) × 100 (2)

where W_L_ is the dry weight of lignin and Ww is the dry weight of extractive-free lignocellulose.

### 4.3. Cellulose Purification

Cellulose content was determined using the Kürschner–Höffer method [77]. Briefly, 0.5 g of extractive-free lignocellulose were refluxed with 25 mL of HNO_3_/ethanol (1:4, *v*/*v*) for 1 h. The solution was decanted and replaced with fresh reagent, and this procedure was repeated three additional times. During the final cycle, 25 mL of 1% KOH solution were added and refluxed for 30 min.

Samples were filtered through medium-pore filter paper using a porcelain filter and oven-dried at 70 °C for 24 h to constant weight. Cellulose content was calculated as:

*Cellulose* (%) = (W_C_/W_W_) × 100 (3)

where Wc is the dry weight of cellulose and Ww is the dry weight of extractive-free lignocellulose.

### 4.4. Hemicellulose Extraction

Hemicellulose extraction was performed following Li et al. [78]. From extractive-free lignocellulose, 0.5 g were refluxed with 10 mL of distilled water for 3 h (solid–liquid ratio 1:20, g/mL). The mixture was cooled, filtered, and the filtrate concentrated to 1.25 mL. Subsequently, 3.75 mL of ethanol (95%) were added, and the mixture was left undisturbed for 1 h to allow for hemicellulose precipitation.

Samples were centrifuged at 4500× *g* for 4 min and dried at 60 °C for 16 h to obtain the initial hemicellulose fraction (H_0_). The insoluble residue was then subjected to successive extractions with KOH solutions (0.6, 1.0, 1.5, 2.0, and 2.5%) at 75 °C for 3 h each (1:20, g/mL). In the final extraction, ethanol (99.7%) was added at a 2:3 ratio. All extracts were filtered, acidified to pH 5.5 with glacial acetic acid, concentrated to 1.25 mL, centrifuged, and dried at 70 °C for 12 h.

The constant weight of each fraction (H_0.6_–H_2.5_) was recorded, and total hemicellulose content was calculated as:

Hemicellulose (%) = (W_H_/W_W_) × 100 (4)

where Wh is the sum of all hemicellulose fractions and Ww is the dry weight of extractive-free lignocellulose.

### 4.5. FTIR Analysis

The syringyl/guaiacyl (S/G) ratio was determined by Fourier-transform infrared (FTIR) spectroscopy following Maceda et al. [12]. Klason lignin samples (0.1 g) were oven-dried at 90 °C for 12 h to remove residual moisture. For each sample, ten FTIR spectra were recorded using an Agilent Cary 630 FTIR spectrometer (30 scans, 4 cm^−1^ resolution, 15 s per scan). Spectra were averaged, baseline-corrected, and deconvoluted to resolve overlapping bands using MicroLab PC software version 5.7 (Agilent Technologies, Santa Clara, CA, USA), following Popescu and Popa [79].

The fingerprint region (800–1800 cm^−1^) was analyzed. Absorption bands at 1269–1272 cm^−1^ and 1328–1330 cm^−1^ were used to quantify guaiacyl (G) and syringyl (S) units, respectively, according to Pandey [80]. The S/G ratio was calculated as the ratio of the intensities of the S and G bands [12].

Crystalline cellulose was analyzed using purified cellulose samples. FTIR spectra were recorded in the 4000–650 cm^−1^ range (30 scans, 4 cm^−1^ resolution, 15 s per scan) and averaged using Resolution Pro FTIR software version 5.4.1 (Agilent Technologies, Santa Clara, CA, USA). The total crystallinity index (TCI) was calculated as the ratio between the intensities of the absorption bands at 1370 and 2900 cm^−1^ [81,82,83].

### 4.6. Leaf Anatomy

The fixed leaf fragments were rinsed with distilled water and stored in 50% ethanol until sectioning. Transverse sections were obtained using a sliding microtome and examined using autofluorescence and staining with acridine orange and Congo red, which bind preferentially to lignin and cellulose, respectively [11,84,85]. These staining and autofluorescence conditions were selected to visualize the spatial distribution of lignified and non-lignified tissues, considering their biochemical fluorescence properties, rather than to perform quantitative comparisons among species.

Micrographs were captured using an epifluorescence microscope equipped with a dichroic cube and three excitation/emission filter sets: DAPI (excitation 365 nm, emission 445/50 nm), FITC (excitation 470/40 nm, emission 525/50 nm), and TRITC (excitation 546/12 nm, emission 575–640 nm). Imaging was performed using a Zeiss Axio Imager Z2 microscope with an AxioCam MRc 5 camera and a metal-halide fluorescence light source (Zeiss HXP 120). To minimize photobleaching, samples were exposed to excitation light for a maximum of 5 min [86]. Brightness and contrast adjustments were applied uniformly using Zen Blue 2.5 lite software (Zeiss, Oberkochen, Germany). For anatomical analysis, multiple transverse sections were obtained from each selected leaf and examined microscopically. The sections presented in the figures correspond to representative samples that illustrate the main anatomical features observed for each species.

### 4.7. Statistical Analysis

All data were tested for normality using the Kolmogorov–Smirnov and Shapiro–Wilk tests. As normality was not met, even after arcsine square-root transformation, non-parametric analyses were applied. Differences among species were evaluated using the Kruskal–Wallis test followed by Dunn’s post hoc test. Given the number of variables analyzed, a principal component analysis (PCA) was conducted to assess species grouping based on structural components. Additionally, cluster analysis was performed to confirm the groupings identified by PCA.

## 5. Conclusions

This study demonstrates that leaves of Asparagaceae species differ markedly in extractive content, lignocellulosic composition, cellulose crystallinity, and lignin monomer composition. *Agave* species were characterized by higher cellulose content and crystallinity, whereas *Yucca* species showed elevated lignin percentages and, in *Y. periculosa*, a higher syringyl contribution.

These chemical traits were closely linked to leaf anatomy, particularly the proportion of non-lignified parenchyma, the distribution of vascular bundles, and the degree of cell wall lignification. Such structural organization reflects adaptive strategies to water-limited environments while also determining the functional properties of the biomass. This integrative assessment of leaf lignocellulosic biomass in Asparagaceae species has not been previously reported.

The predominance of crystalline cellulose suggests suitability for fiber-based biomaterials and cellulose-derived products, whereas high guaiacyl-type lignin content supports potential applications in bioenergy and carbon-rich materials. By providing the first comparative chemical–anatomical dataset for leaves of multiple Asparagaceae genera, this study contributes novel information relevant to the sustainable exploitation of drought-adapted plants as non-food lignocellulosic resources.

## Figures and Tables

**Table 1:** Chemical composition of leaf biomass from selected Asparagaceae species, including extractives, lignin, cellulose, and hemicellulose contents. Values are expressed as a percentage of dry weight and represent mean ± standard deviation (n = 3).

**Extractives**				
**Species**	**Ethanol:Toluene**	**Ethanol 96%**	**Water 90 °C**	**Total Extractives**
*A. convallis* Trel.	12.46 ± 1.19 BC	2.04 ± 0.51 A	4.20 ± 0.33 ABC	18.7 ± 0.95 C
*A. salmiana* Otto ex. Salm-Dyck	11.17 ± 1.62 BC	4.62 ± 0.84 B	8.21 ± 0.85 E	24.00 ± 0.85 D
*A. striata* Zucc.	9.92 ± 0.33 AB	1.36 ± 0.20 A	4.32 ± 0.67 BC	15.60 ± 0.97 B
*D. acrotrichum* Zucc.	18.26 ± 0.88 D	2.62 ± 1.40 A	2.68 ± 0.70 AB	23.56 ± 0.5 D
*N. excelsa* García-Mend. & E.Solano	13.48 ± 1.83 C	2.44 ± 0.39 A	2.46 ± 0.13 A	18.39 ± 1.47 C
*Y. filifera* Chabaud	7.98 ± 1.19 A	1.18 ± 0.22 A	6.54 ± 1.27 D	15.70 ± 0.84 B
*Y. periculosa* Baker	7.09 ± 0.13 A	0.92 ± 0.11 A	5.36 ± 0.37 CD	13.37 ± 0.5 A
**Lignocellulose**				
**Species**	**Lignocellulose**	**Lignin**	**Cellulose**	**Hemicellulose**
*A. convallis*	81.30 ± 0.95 B	29.08 ± 1.22 AB	41.09 ± 1.23 C	29.83 ± 2.15 B
*A. salmiana*	76.00 ± 0.85 A	28.57 ± 0.91 A	42.15 ± 0.66 C	29.28 ± 0.40 B
*A. striata*	84.40 ± 0.97 C	32.12 ± 1.24 BC	31.56 ± 0.92 A	36.32 ± 2.00 C
*D. acrotrichum*	76.44 ± 0.51 A	34.11 ± 1.03 CD	35.06 ± 1.44 B	30.83 ± 0.45 B
*N. excelsa*	81.61 ± 1.47 B	36.84 ± 0.68 D	34.58 ± 1.02 B	28.58 ± 0.41 B
*Y. filifera*	84.30 ± 0.84 C	45.07 ± 1.35 E	31.35 ± 0.82 A	23.57 ± 1.44 A
*Y. periculosa*	86.63 ± 0.51 D	42.79 ± 2.71 E	33.81 ± 0.83 AB	23.40 ± 3.34 A

Different letters in each column indicate significant differences (*p* < 0.05). Mean ± standard deviation.

**Table 2:** Total crystallinity index (TCI) of leaf lignin and cellulose from selected Asparagaceae species, determined by FTIR spectroscopy. TCI was calculated from the ratio of band intensities at 1370 and 2900 cm^−1^.

Species	Crystalline %	Amorphous %	TCI (A_1370_/A_2900_)
*A. convallis*	56.9	43.1	1.32
*A. salmiana*	53.5	46.5	1.15
*A. striata*	45.3	54.7	0.83
*D. acrotrichum*	46.9	53.1	0.88
*N. excelsa*	52.9	47.1	1.12
*Y. filifera*	54.6	45.4	1.20
*Y. periculosa*	42.1	57.9	0.73

**Table 3:** Syringyl/guaiacyl (S/G) ratio of leaf lignin and cellulose from selected Asparagaceae species, determined by FTIR spectroscopy. The S/G ratio was calculated from the relative intensities of absorption bands at 1325 and 1271 cm^−1^.

Species	S %	G %	S/G
*A. convallis*	45.3	54.7	0.83
*A. salmiana*	46.2	53.8	0.86
*A. striata*	46.8	53.2	0.88
*D. acrotrichum*	46.9	53.1	0.88
*N. excelsa*	46.8	53.2	0.88
*Y. filifera*	49.3	50.7	0.98
*Y. periculosa*	54.6	45.4	1.2

**Table 4:** PCA for chemical and structural variables of leaves from seven Asparagaceae species. Variables with higher values have a greater influence on species separation in multivariate space.

Variables	PC1	PC2	PC3
Ethanol:Toluene	0.326	0.016	**−0.679**
Ethanol	−0.417	−0.453	0.015
Hot water	0.319	−0.035	**0.666**
Extractive-free lignocellulose	0.038	**0.657**	0.232
Cellulose	0.333	**−0.526**	0.158
Lignin	**−0.541**	0.222	−0.020
Hemicellulose	**0.462**	0.189	−0.125
Eigenvalue	2.780	1.895	1.222
Variance (%)	39.71	27.07	17.45
Accumulative variance (%)	39.71	66.77	84.23

Bold numbers indicate the variables that most strongly contributed to species separation.

**Table 5:** Asparagaceae species.

Species	Life Forms	Code
*A. convallis* Trel.	Herbaceous	JBAgcon
*A. salmiana* Otto ex. Salm-Dyck	Herbaceous	TT8810
*A. striata* Zucc.	Herbaceous	JBAgst
*D. acrotrichum* Zucc.	Shrubby	JBDasy
*N. excelsa* García-Mend. & E.Solano	Arborescent	JBNoex
*Y. filifera* Chabaud	Arborescent	JBYufi
*Y. periculosa* Baker	Arborescent	JBYupe

## Data Availability

The original data presented in the study are openly available in Zenodo at https://doi.org/10.5281/zenodo.18295631 (accessed on 29 January 2026).

## Supplementary Materials

The following supporting information can be downloaded at: https://www.mdpi.com/article/10.3390/plants15030468/s1, Table S1: Percentages of lignocellulose compounds of Asparagaceae species. Table S2. Collection number of the seven Asparagaceae species from the Botanical Garden of UNAM.
