# Supplementary material for: Chemical Composition and Structural Traits of Leaf Biomass in Selected Asparagaceae Species
Source: Plants (Basel). 2026 Feb 2;15(3):468. doi: 10.3390/plants15030468 (PMC12899159; doi:10.3390/plants15030468)
Supplement: Supplementary file 1 [file plants-15-00468-s001.zip › plants-4094737-supplementary.pdf]

Supplementary material

Table S1. Summary of anatomical traits measured in leaves of the studied Asparagaceae species, including tissue proportions and cell wall characteristics. All abbreviations are defined within the table.

Species	Sample	Ce	He	Li	Ref
A. americana	L	68.4	15.7	4.9	[1]
	St	65	32	3	[2]
	L	50	22	13	[3]
	L, St	-	-	8.2	[4]
A. angustifolia	F	55.03	34.08	20.69	[5]
	F	67	25.2	6.3	[3]
	F	64	25	6.5	[6]
A. fourcroydes	L	77.6	5 -7	13.1	[7]
A. lechuguilla	St	17.72	17.5	7.32	[8]
	St	-	-	9.07	[9]
	L	46-48	30	11	[10]
	L	79.8	3-6	15.3	[7]
A. salmiana	F	48.94	-	8.48	[11]
	B	47.3	12.8	4.9	[12]
	L, St	-	-	9.8	[4]
A. sisalana	-	43	32	15	[13]
	F	49.43	-	21.1	[14]
	F	41.9	-	7.2	[15]
A. tequilana	L, St	40 and 51	-	-	[16]
	B	56-69	-	-	[17]
	B	40.94	-	-	[18]
	B	42	18.5	14	[19]
	B	-	-	-	[20]
	B	44.5	20.1	25.3	[21]
	L	24.7-33.5	10.7-15.2	15.6-19.5	[22]
	L	47	16	9	[23]
	L, St	-	-	11.9	[4]
Furcraea foetida	F	68.35	11.46	12.32	[24]
Yucca aloifolia	L	52.5	20.5	20	[25]
Yucca gloriosa	F	66.4	17.5	6.7	[26]
A. angustifolia, A. lechuguilla, A. salmiana, A. tequilana	L	33.23-44.25	2.57-3.47	2.11-2.86	[27]
A. salmiana, A. americana, A. tequilana	L, St	39.7-45		7.3-11.9	[28]

Ce: cellulose, He: hemicellulose, Li: lignin, Ref: references. *B: Bagasse, F: Fiber, L: Leaf, St: Stem. Data were compiled from the literature to provide contextual comparison with the experimental results obtained in the present study.

Table S2. Collection number of the seven Asparagaceae species from the Botanical Garden of UNAM.

Species	Collection number*
A. convallis Trel.	AGM7308
A. salmiana Otto ex. Salm-Dyck	TT1086
A. striata Zucc.	AGM7860
D. acrotrichum Zucc.	AGM7369
N. excelsa García-Mend. & E.Solano	TT1088
Y. filifera Chabaud	AGM7221
Y. periculosa Baker	AGM6332

*The collection number corresponds to the material reserved in the MEXU herbarium

References

1. Mylsamy, K.; Rajendran, I. Investigation on Physio-chemical and Mechanical Properties of Raw and Alkali-treated Agave americana Fiber. *J. Reinf. Plast. Compos.* **2010**, *29*, 2925–2935, doi:10.1177/0731684410362817.

2. Krishnadev, P.; Subramanian, K.S.; Janavi, G.J.; Ganapathy, S.; Lakshmanan, A. Synthesis and Characterization of Nano-fibrillated Cellulose Derived from Green Agave americana L. Fiber. *BioResources* **2020**, *15*, 2442–2458.

3. Rosli, N.A.; Ahmad, I.; Abdullah, I. Isolation and characterization of cellulose nanocrystals from Agave angustifolia fibre . *BioResources* **2013**, *8*, 1893–1908.

4. Li, H.; Pattathil, S.; Foston, M.B.; Ding, S.Y.; Kumar, R.; Gao, X.; Mittal, A.; Yarbrough, J.M.; Himmel, M.E.; Ragauskas, A.J.; et al. Agave proves to be a low recalcitrant lignocellulosic feedstock for biofuels production on semi-arid lands. *Biotechnol. Biofuels* **2014**, *7*, 50, doi:10.1186/1754-6834-7-50.

5. Hidalgo-Reyes, M.; Caballero-Caballero, M.; HernáNdez-Gómez, L.H.; Urriolagoitia-Calderón, G. Chemical and morphological characterization of Agave angustifolia bagasse fibers. *Bot. Sci.* **2015**, *93*, 807–817, doi:10.17129/botsci.250.

6. Teli, M.D.; Jadhav, A.C. Effect of alkali treatment on the properties of Agave augustifolia v. marginata fibre. *Int. Res. J. Eng. Technol.* **2016**, *03*, 2754–2761.

7. Vieira, M.C.; Heinze, T.; Antonio-Cruz, R.; Mendoza-Martinez, A.M. Cellulose derivatives from cellulosic material isolated from Agave lechuguilla and fourcroydes. *Cellulose* **2002**, *9*, 203–212, doi:10.1023/A:1020158128506.

8. Carmona, J.E.; Morales-Martínez, T.K.; Mussatto, S.I.; Castillo-Quiroz, D.; Ríos-González, L.J. Chemical, structural and functional properties of lechuguilla (Agave

lechuguilla Torr.). *Rev. Mex. ciencias For.* **2017**, *8*, 100–122.

9. Ortíz-Méndez, O.H.; Morales-Martínez, T.K.; Rios-González, L.J.; Rodríguez-De La Garza, J.A.; Quintero, J.; Aroca, G. Bioethanol production from Agave lechuguilla biomass pretreated by autohydrolysis. *Rev. Mex. Ing. Química* **2017**, *16*, 467–476.

10. Márquez, A.; Cazaurang, N.; González, I.; Colunga-GarciaMarin, P. Extraction of chemical cellulose from the fibers of Agave lechuguilla Torr. *Econ. Bot.* **1996**, *50*, 465–468.

11. De Dios Naranjo, C.; Alamilla-Beltrán, L.; Gutiérrez-Lopez, G.F.; Terres-Rojas, E.; Solorza-Feria, J.; Romero-Vargas, S.; Yee-Madeira, H.T.; Areli, F.-M.; Mora-Escobedo, R. Aislamiento y caracterización de celulosas obtenidas de fibras de Agave salmiana aplicando dos métodos de extracción ácido-alcali*. *Rev. Mex. Ciencias Agrícolas* **2016**, *7*, 31–43.

12. Bernardo, G.R.R.; Rene, R.M.J. Contribution of agro-waste material main components (hemicelluloses, cellulose, and lignin) to the removal of chromium (III) from aqueous solution. *J. Chem. Technol. Biotechnol.* **2009**, *84*, 1533–1538, doi:10.1002/JCTB.2215.

13. McDougall, G.J.; Morrison, I.M.; Stewart, D.; Weyers, J.D.B.; Hillman, J.R. Plant fibres: Botany, chemistry and processing for industrial use. *J. Sci. Food Agric.* **1993**, *62*, 1–20, doi:10.1002/JSFA.2740620102.

14. Kestur G., S.; Flores-Sahagun, T.H.S.; Dos Santos, L.P.; Dos Santos, J.; Mazzaro, I.; Mikowski, A. Characterization of blue agave bagasse fibers of Mexico. *Compos. Part A Appl. Sci. Manuf.* **2013**, *45*, 153–161, doi:10.1016/J.COMPOSITESA.2012.09.001.

15. Iñiguez, G.; Acosta, N.; Martinez, L.; Parra, J.; González, O. Utilización de supbroductos de la industria tequilera. Parte 7. Compostaje de bagazo de agave y vinazas tequileras. *Rev. Int. Contam. Ambient.* **2005**, *21*, 37–50.

16. Robles, E.; Fernández-Rodríguez, J.; Barbosa, A.M.; Gordobil, O.; Carreño, N.L. V; Labidi, J. Production of cellulose nanoparticles from blue agave waste treated with environmentally friendly processes. *Carbohydr. Polym.* **2018**, *183*, 294–302, doi:10.1016/j.carbpol.2018.01.015.

17. Hernández, J.A.; Romero, V.H.; Escalante, A.; Toriz, G.; Rojas, O.J.; Sulbarán, B.C. Agave tequilana bagasse as source of cellulose nanocrystals via organosolv treatment. *BioResources* **2018**, *13*, 3603–3614.

18. Robles-García, M.Á.; Del-Toro-Sánchez, C.L.; Márquez-Ríos, E.; Barrera-Rodríguez, A.; Aguilar, J.; Aguilar, J.A.; Reynoso-Marín, F.J.; Ceja, I.; Dórame-Miranda, R.; Rodríguez-Félix, F. Nanofibers of cellulose bagasse from Agave tequilana Weber var. azul by electrospinning: preparation and characterization. *Carbohydr. Polym.* **2018**, *192*, 69–74, doi:10.1016/J.CARBPOL.2018.03.058.

19. Ramírez-Cortina, C.; Alonso-Gutiérrez, M.S.; Rigal, L. Tratamiento alcalino de los residuos agroindustriales de la producción del tequila, para su uso como complemento de alimento de rumiantes. *Rev. AIDIS Ing. y ciencias Ambient.* **2012**, *5*, 69–77.

20. Espino, E.; Cakir, M.; Domenek, S.; Román-Gutiérrez, A.D.; Belgacem, N.; Bras, J.

Isolation and characterization of cellulose nanocrystals from industrial by-products of Agave tequilana and barley. *Ind. Crops Prod.* **2014**, *62*, 552–559, doi:10.1016/J.INDCROP.2014.09.017.

21. Palacios Hinestroza, H.; Hern ́, J.A.H.; Diaz, H.; Alfaro, M.E.; Toriz, G.; Rojas, O.J.; Sulbarán-Rangel, B.C. Isolation and Characterization of Nanofibrillar Cellulose from Agave tequilana Weber Bagasse. *Adv. Mater. Sci. Eng.* **2019**, *2019*, 1342547, doi:10.1155/2019/1342547.

22. Rijal, D.; Walsh, K.B.; Subedi, P.P.; Ashwath, N. Quality Estimation of Agave Tequilana Leaf for Bioethanol Production. *http://dx.doi.org/10.1255/jnirs.1247* **2017**, *24*, 453–465, doi:10.1255/JNIRS.1247.

23. Corbin, K.R.; Byrt, C.S.; Bauer, S.; Debolt, S.; Chambers, D.; Holtum, J.A.M.; Karem, G.; Henderson, M.; Lahnstein, J.; Beahan, C.T.; et al. Prospecting for Energy-Rich Renewable Raw Materials: Agave Leaf Case Study. *PLoS One* **2015**, *10*, e0135382, doi:10.1371/JOURNAL.PONE.0135382.

24. Manimaran, P.; Senthamaraikannan, P.; Sanjay, M.R.; Marichelvam, M.K.; Jawaid, M. Study on characterization of Furcraea foetida new natural fiber as composite reinforcement for lightweight applications. *Carbohydr. Polym.* **2018**, *181*, 650–658, doi:10.1016/J.CARBPOL.2017.11.099.

25. do Nascimento, H.M.; dos Santos, A.; Duarte, V.A.; Bittencourt, P.R.S.; Radovanovic, E.; Fávaro, S.L. Characterization of natural cellulosic fibers from Yucca aloifolia L. leaf as potential reinforcement of polymer composites. *Cellulose* **2021**, *28*, 5477–5492, doi:10.1007/S10570-021-03866-Y.

26. Taban, E.; Mirzaei, R.; Faridan, M.; Samaei, E.; Salimi, F.; Tajpoor, A.; Ghalenoei, M. Morphological, acoustical, mechanical and thermal properties of sustainable green Yucca (Y. gloriosa) fibers: an exploratory investigation. *J. Environ. Heal. Sci. Eng.* **2020**, *18*, 896, doi:10.1007/S40201-020-00513-9.

27. Jiménez-Muñóz, E.; Prieto-García, F.; Prieto-Méndez, J.; Acevedo-Sandoval, O.A.; Rodríguez-Laguna, R. Physicochemical characterization of four species of agaves with potential in obtaining pulp for paper making. *DYNA* **2016**, *83*, 232–242, doi:10.15446/dyna.v83n197.52243.

28. Li, H.; Foston, M.B.; Kumar, R.; Samuel, R.; Gao, X.; Hu, F.; Ragauskas Cd, A.J.; Wyman, C.E. Chemical composition and characterization of cellulose for Agave as a fast-growing, drought-tolerant biofuels feedstock. *RSC Adv.* **2012**, *2*, 4951–4958, doi:10.1039/c2ra20557b.
